# Sequencing and analysis of the complete mitochondrial genome of *Eothenomys eleusis* Thomas 1911 from China and its phylogenetic analysis

**DOI:** 10.1080/23802359.2023.2197087

**Published:** 2023-04-10

**Authors:** Liu Zhu, Zhong-Dan Bi, Hong-An Qian, Xiu-Feng Mei, Jing-Yu Zhao, Xin-Xu Zhao, Huan Chen, Jun-Sheng Zhang, Zhong-Wan Piao

**Affiliations:** College of Life Science and Technology, Mudanjiang Normal University, Mudanjiang, P.R. China

**Keywords:** Mitogenome, phylogenetic trees, *Eothenomys eleusi*

## Abstract

The complete mitogenome sequence of *Eothenomys eleusis* Thomas 1911 was determined using PCR. A circular double-stranded structure makes up the mitochondrial genome of *E. eleusis*. The complete length of the mitochondrial genome is 16,419 bp. The mitochondrial genome of *E. eleusis* included 13 protein-coding genes, 1 control region, 22 tRNA genes, 2 rRNA genes and 1 origin of L strand replication. The total base composition of *E. eleusis* mitochondrial genome was A (32.6%), T (26.3%), G (13.6%) and C (27.5%). We found significant A-T skew in base composition, especially in control regions and protein-coding genes. *E. eleusis* was supported by bootstrap values of 100%. This study verifies the evolutionary status of *E. eleusis* in Myodini tribe of Cricetidae at the molecular level. The mitochondrial genome would be a significant supplement for the *E. eleusis* genetic background.

## Introduction

*Eothenomys eleusis* Thomas 1911 belongs to the Rodentia order, Cricetidae family, Arvicolinae subfamily, Myodini tribe, genus *Ethenomys* (Wilson and Reeder et al. 2005). The species of the genus *Ethenomys* is relatively abundant in China (Liu et al. 2019). The genus is currently thought to have 17 species in China (Wei et al. 2021). *E. eleusis* is endemic to China (Wei et al. 2021). *E. eleusis* is widely distributed in south China (Liu et al. 2019). The taxonomy and phylogeny of Myodini tribe, especially in *Eothenomys*, have been controversial. In this study, the complete mitochondrial genome of *E. eleusis* was sequenced, and the phylogenetic relationships within Myodini tribe were analyzed.

## Material & methods

**Figure 1.**
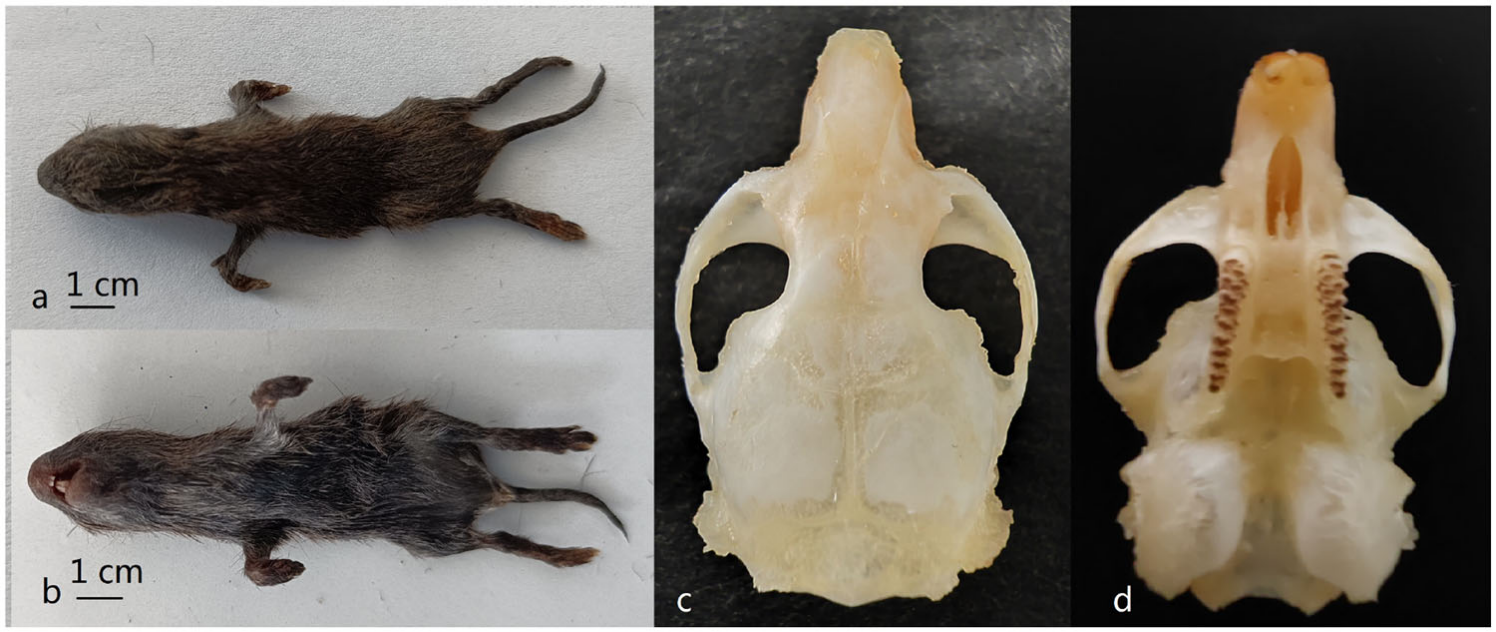
The picture of external morphology and skull morphology. a: External morphology on the back, b: External morphology on the belly, c: Dorsal view of the maxilla, d: Ventral view of the maxilla. The morphological characters external morphology and skull morphology: Head and body length is 90 mm, Tail length is 42 mm, Tail length is about 45% of Head and body length, the color of back the body is black-brown, the molars have on root, the chewing surface of molars is wide. The specimen was collected by Liu zhu from the Anshun City (26°21′48″N, 105°55′35″E), in Guizhou Province, China, in August 2021. This picture was taken by Liu zhu.

A dead female *E. eleusis* was collected from Anshun City (26°21′48″N, 105°55′35″E), in Guizhou Province, China, in August 2021. This study got permission from the Laboratory Animal Welfare Ethics Committee of Mudanjiang Normal University. We described and measured the external morphology and skull morphology of the specimen (Figure 1). We distinguished species using the key of Myodini in China (Tang et al. 2021). The *Cyt b* gene sequence of the specimen was blast in Genbank. The sequence (HM165380) the most similar to the specimen is from *E. eleusis*. The sample was stored at −75 °C before being used. The specimen was deposited at the Animal and Plant Herbarium of Mudanjiang Normal University (URL, Liu zhu and swxlz0@126.com) under the voucher number KQRS2021004. Genomic DNA was extracted from leg muscle using the EasyPure genomic DNA kit (TransGen Biotech Co., Beijing, China). Based on the reported mitochondrial genome of *Eothenomys*, we designed 15 pairs of primers for PCR. PCR gel images of 15 pairs of primers were taken (Figure S1). The first-generation sequencing technology was used in the sequencing of this study (the ABI 3730 sequencer, Boshi Biotechnology Co. Ltd., Haerbin, China). The sequences were assembled using DNA star, and analyzed and adjusted manually. The annotation of the *E. eleusis* mitochondrial genome was fulfilled using web-based services MITOS (http://mitos.bioinf.uni-leipzig.de/help.py) and software PhyloSuite v 1.2.2 (Zhang et al. 2020). The circular mitochondrial genome map of *E. eleusis* was drawn using OGDRAW 1.3.1 (Greiner et al. 2019). In this study, the molecular phylogeny of *E. eleusis* was investigated using the complete mitochondrial genomes of 14 species from 5 genera in Myodini tribe deposited in the GenBank. The phylogenetic tree was constructed by the nucleotide sequences of the complete mitochondrial genome without partitioning, through MEGA 11.0 software (Tamura et al. 2011). The phylogenetic tree was constructed using the Kimura 2-parameter model of Maximum Likelihood method with 1000 bootstrap replications.

## Results

**Figure 2.**
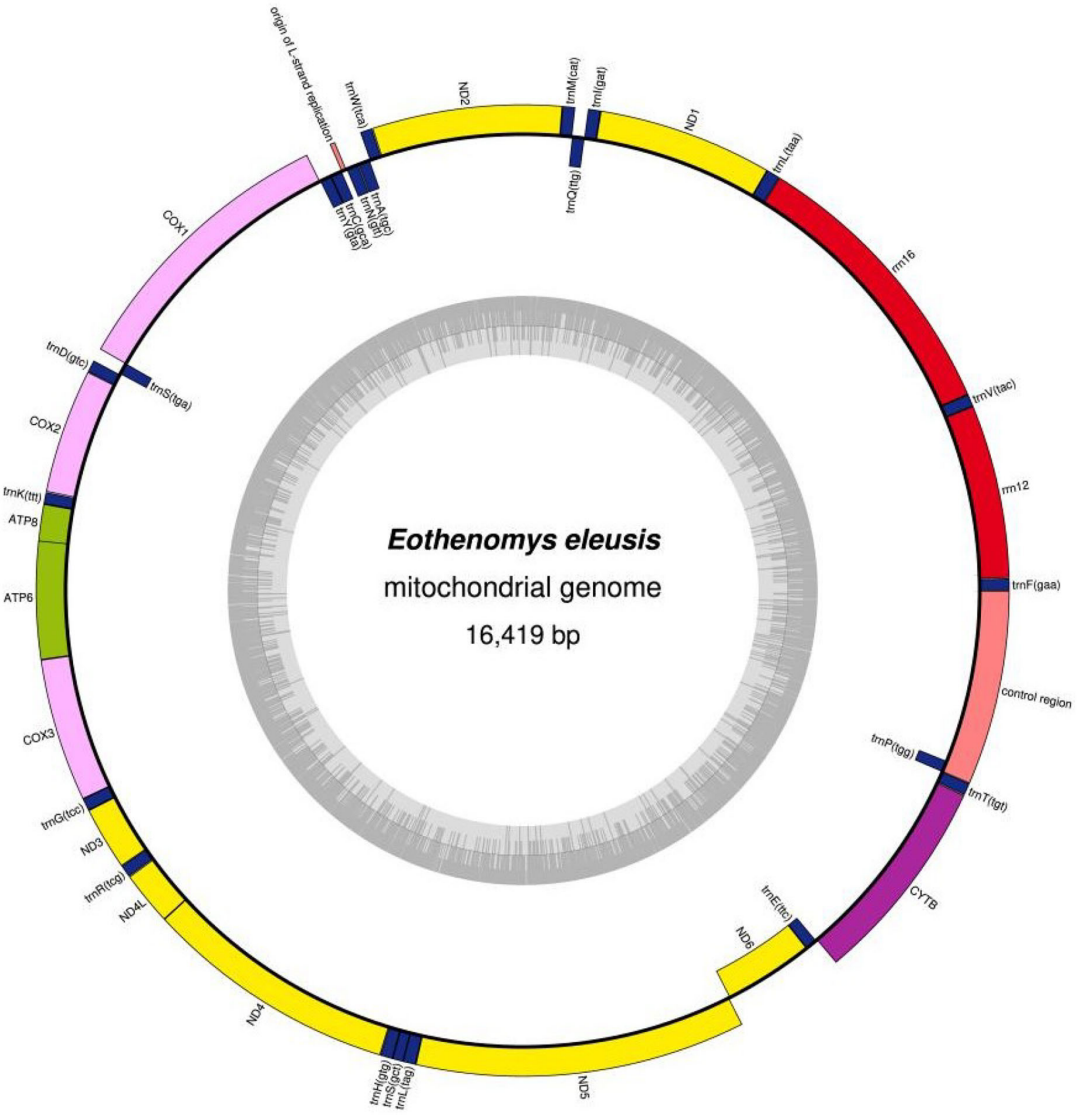
The circular mitochondrial genome map of *E. Eleusis.* The circular inside is L strand, and the circular outside is H strand. Yellow: *NADH* gene, pink: *COX* gene, green: *ATP* gene, purple: other genes, blue: tRNA, red: rRNA, nude: origin of L strand replication and control region.

**Figure 3.**
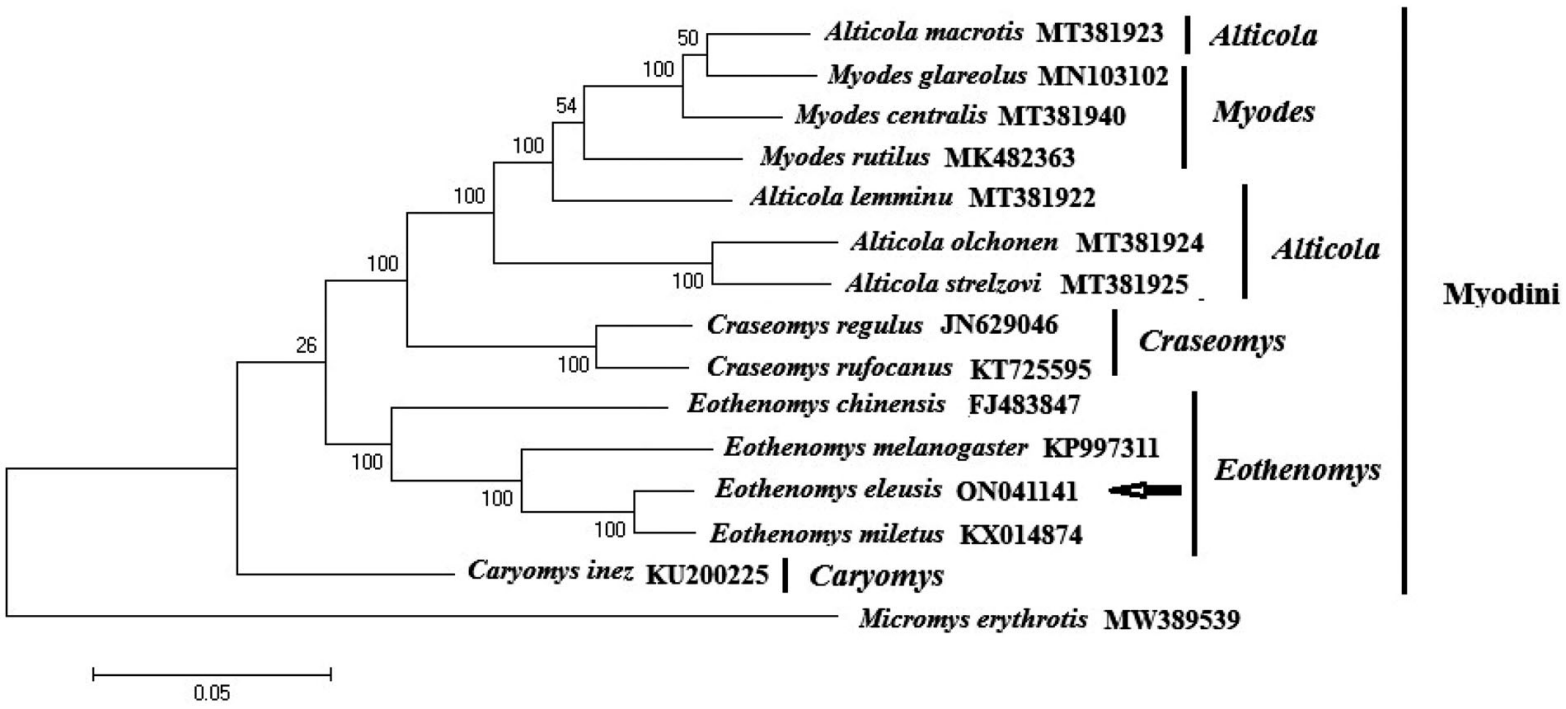
The phylogenetic tree constructed by the nucleotide sequences of complete mitochondrial genome without partitioning, through MEGA 11.0 software. The phylogenetic tree was constructed using the Kimura 2-parameter model of Maximum Likelihood method with 1000 bootstrap replications. *Alticola strelzovi* (Abramson et al. 2021), *Alticola olchonensis* (Abramson et al. 2021), *Alticola lemminus* (Abramson et al. 2021), *Alticola macrotis* (Abramson et al. 2021), *Eothenomys miletus* (Mu et al. 2019), *Eothenomys melanogaster* (Chen et al. 2016), *Eothenomys chinensis* (Yang et al. 2012), *Eothenomys eleusis*, *Craseomys regulus* (Abramson et al. 2021), *Craseomys rufocanus* (Lu et al. 2017), *Myodes rutilus* (Jin et al. 2019), *Myodes centralis* (Abramson et al. 2021), *Myodes glareolus* (Markováet et al. 2020), *Caryomys inez* (Yu et al. 2016). The out group is *Micromys erythrotis* (Cai et al. 2021).

A circular double-stranded structure makes up the mitochondrial genome of *E. eleusis* (Figure 2). The complete length of the mitochondrial genome is 16,419 bp. The mitochondrial genome of *E. eleusis* included 13 protein-coding genes, 1 control region, 22 tRNA genes, 2 rRNA genes and 1 origin of L strand replication (Figure 2). The total base composition of *E. eleusis* mitochondrial genome was A (32.6%), T (26.3%), G (13.6%) and C (27.5%). We found significant A-T skew in base composition, especially in control regions and protein-coding genes. The *ND6* gene and 8 tRNA genes of *E. eleusis* were encoded on the L strand. The other mitochondrial genes were encoded on the H strand (Figure 2). GenBank received the annotated mitochondrial genome sequences, and the accession number ON041141 was given. Between the tRNA-Pro and tRNA-Phe genes is the control region of the mitochondrial genome (Figure 2). The control region has no structural genes, but has only promoters and regulatory sequences for replication and transcription. The total length of 13 protein-coding genes sequences is 11,395 bp. The start codon for most protein-coding genes is ATG. The start codons for *ND1*, *ND2* and *ND3* is ATA or ATT. The stop codon for nine protein-coding genes is TAA, while *ND4*, *COX3* use the incomplete stop codons (T– –). The other stop codons are TAG. The length of 22 tRNA genes are between 59 and 75 bp. The length of L-strand replication origin (*OL*) was 30 bp. On phylogenetic tree (Figure 3), the 4 genera (*Craseomys*, *Caryomys*, *Myopus* and *Eothenomys*) in Myodini tribe formed independent branches. *Alticola macrotis* and *A. lemminus of Alticola* genus clustered on a branch with the *Myopus* genus (Figure 3). *E. eleusis* was supported by bootstrap values of 100% (Figure 3). Our results show that *E. eleusis* and *E. miletus* have a closer phylogenetic relationship (Figure 3).

## Discussion & conclusions

The arrangement of genes in *E. eleusis* mitochondrial genome is consistent with other Cricetidae species (Chen et al. 2016; Cong et al. 2016; Luo and Liao 2016; Zhang et al. 2016; Jiang et al. 2018; Abramson et al. 2021; Baca et al. 2021). The phylogenetic relationship of this study is basically consistent with that of the recently published literature on Arvicolinae subfamilies (Liu et al. 2019; Abramson et al. 2021). This study verifies the evolutionary status of *E. eleusis* in Myodini tribe of Cricetidae at the molecular level. The mitochondrial genome would be a significant supplement for the *E. eleusis* genetic background.

## Supplementary Material

Supplemental Material

## Data Availability

The data that support the findings of this study are openly available in GenBank at https://www.ncbi.nlm.nih.gov/, reference number ON041141.
