# Supplementary material for: Sequencing and analysis of the complete mitochondrial genome of Eothenomys eleusis Thomas 1911 from China and its phylogenetic analysis
Source: Mitochondrial DNA B Resour. 2023 Apr 10;8(4):493–6. doi: 10.1080/23802359.2023.2197087 (PMC10088922; doi:10.1080/23802359.2023.2197087)
Supplement: Supplemental Material [file TMDN_A_2197087_SM0629.doc]

Supplementary materials


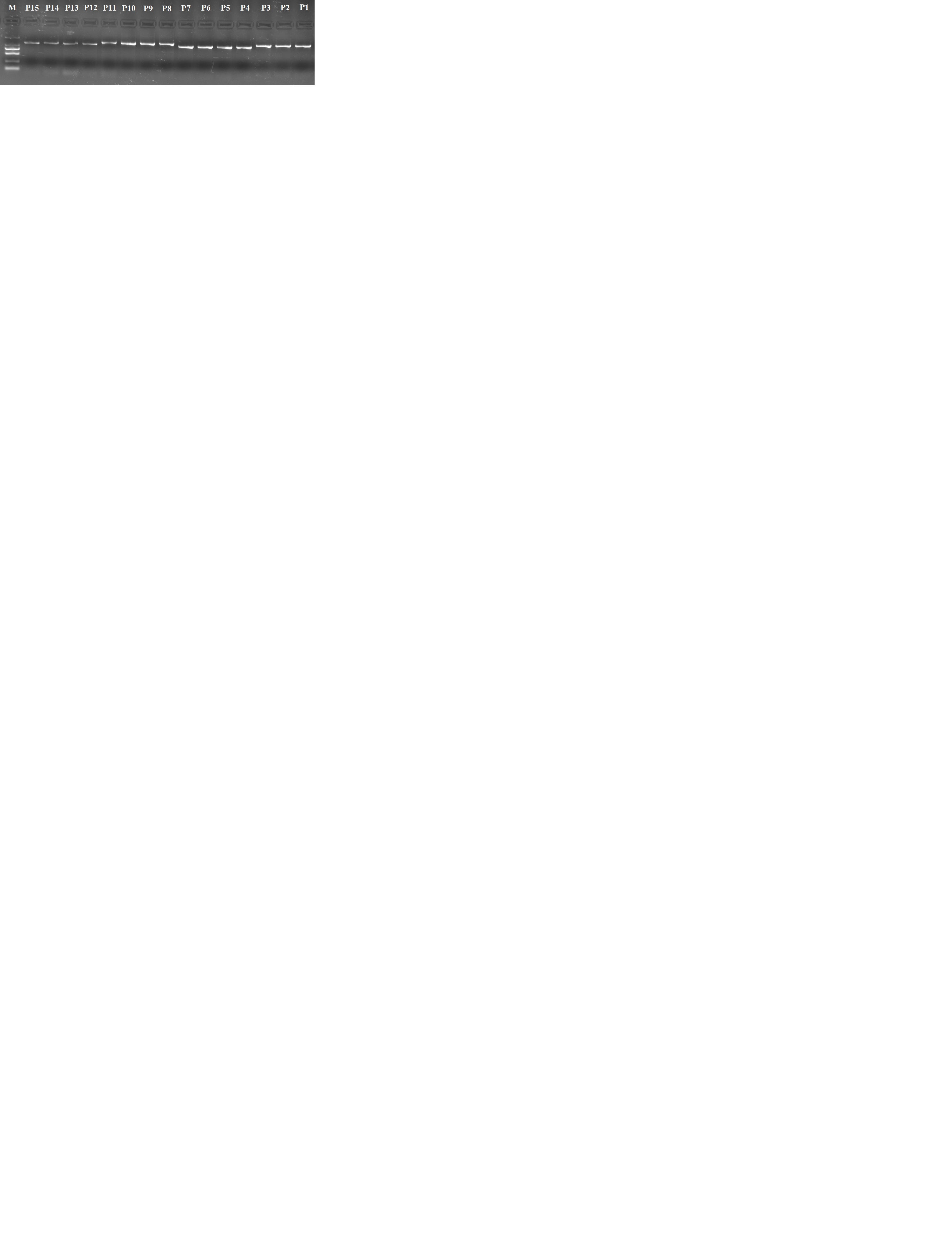


Figure S1. PCR gel images of 15 pairs of primers. Based on the reported mitochondrial genome of *Eothenomys*, we designed 15 pairs of primers for PCR of the complete mitochondrial genome of *Eothenomys eleusis.*P1-P15 stands for primer 1-primer 15. M stands for marker (DL2000)
